# Reducing wait time from referral to first visit for community outpatient services may contribute to better health outcomes: a systematic review

**DOI:** 10.1186/s12913-018-3669-6

**Published:** 2018-11-20

**Authors:** Annie K. Lewis, Katherine E. Harding, David A. Snowdon, Nicholas F. Taylor

**Affiliations:** 1 Allied Health Clinical Research Office, Eastern Health, Level 2/5 Arnold Street, Box Hill, VIC 3128 Australia; 2 La Trobe University, Bundoora, VIC 3086 Australia

**Keywords:** Waiting lists, Access, Appointments and schedules, Outpatients, Community health, Patient outcomes

## Abstract

**Background:**

Many people wait long periods for community outpatient services. However little is known about the impact of waiting from referral to first visit on patient outcomes. The aim of this systematic review is to investigate whether waiting for community outpatient services is associated with adverse effects on patient outcomes.

**Methods:**

Medline, Embase, Psych Info and CINAHL databases were searched, combining the key concepts of waiting for healthcare and patient outcomes. Studies were included if they reported data comparing health outcomes for patients with different waiting times for the same period. Three reviewers applied inclusion and exclusion criteria to identified studies and assessed quality using the McMaster Critical Review Forms. Levels of evidence were assessed using National Health and Medical Research Council guidelines. Included studies were analysed using a descriptive synthesis, and summarised according to levels of evidence and clinical significance for key outcomes.

**Results:**

Fourteen studies that included 69,606 adult patients were selected. Selected studies included patients referred for treatment for musculoskeletal disorders (*n* = 28,722) or to cardiac rehabilitation (*n* = 40,884). There was low-level evidence that reduced wait time is associated with moderate improvement in workplace participation for patients seeking care for musculoskeletal conditions; and moderate improvement in exercise tolerance for patients referred to cardiac rehabilitation. There was inconsistent evidence that improvements in quality of life, patient satisfaction and psychological symptoms may be associated with shorter wait times. Pain, function and physical activity outcomes were not associated with wait time.

**Conclusions:**

This review found low-level evidence suggesting an association between early access to community outpatient services and improvement of some patient outcomes. Specifically, shorter wait times from referral to first visit for musculoskeletal pain services may improve patient work participation. Shorter wait times for cardiac rehabilitation may improve patient exercise capacity. The effects of a short wait time for other patient conditions and patient outcomes, including quality of life, psychological symptoms and patient experience, are inconclusive. The modest benefits in health outcomes observed in reducing wait time for community outpatient services suggest that other possible benefits such as increasing patient flow should be explored.

**Trial registration:**

PROSPERO registration no: CRD42016047003

## Background

Community outpatient services provide health care for individuals with non-acute health conditions [1, 2]. These services are often provided by allied health professionals either working alone or within multi-disciplinary teams, and deliver care either in the home or centre environment. Community outpatient services are increasingly important in the transition from hospital to home or in helping people to manage their healthcare needs within the community [3]. They also help to reduce pressure on hospital services. The demand on community outpatient services is high, and is growing with the shift in focus from bed-based, hospital treatment to self-management or care supported in the community [4, 5].

Improving patient flow through health systems in an effort to maximise capacity and efficiency has received attention [6], but the emphasis of this work has been largely on acute care settings [7, 8]. In emergency departments, for example, there has been an increasing focus on seeing patients as quickly as possible with policy makers instituting incentives for hospitals to meet wait time targets [9, 10]. However, there has been increasing recognition that healthcare involves complex systems [3], where patient flow in one part of the health service is affected by patient flow in another [11, 12]. Efforts to improve patient flow therefore need to include all parts of the system, including community outpatient services.

As community outpatient services are, by nature, not acute or urgent, wait lists are a common strategy used to manage demand in these settings and can result in long delays for care [13–15]. A wide range of initiatives have been used to improve patient flow and reduce waiting times in outpatient health care settings [11], such as lean approaches, triage and prioritisation, Specific and Timely Assessments for Triage, Advanced Access and rationing [12, 16–18]. Apart from the effect on patient flow through the health network, resources invested in reducing delay in provision of community outpatient services are based on an assumption that waiting has a negative impact on patients. Delayed provision of healthcare may lead to poor patient outcomes such as reduced quality of care, pain, stress and anxiety, and erosion in confidence in the health system [11]. It can be assumed that the health outcomes of some patients with acute medical conditions requiring urgent treatment will suffer without timely access to care, but it is not clear whether this assumption also applies to patients awaiting less urgent care from community outpatient services. Some conditions, such as non-specific low back disorders, may resolve within a few weeks without intervention [19]. For chronic conditions, such as arthritis, it is possible that there is no significant worsening of symptoms, function or psychosocial status if patients wait compared to if they are seen promptly.

Given the efforts and resources required to manage and improve patient flow in health services it is important to know if waiting makes a difference to patients, and whether such differences are clinically significant. This review therefore aimed to investigate whether a delay in access to an ambulatory or community service is associated with poorer patient outcomes. That is, does waiting really matter?

## Methods

### Protocol and registration

This review was registered prospectively with PROSPERO (registration number- CRD42016047003) to answer the following question: Does delay in access to community outpatient services change patient outcomes?

This systematic review was reported according to the Preferred Reporting Items for Systematic Reviews and Meta-Analyses (PRISMA) guidelines [20].

### Search strategy

The search strategy aimed to identify studies that compared outcomes of individuals (adults and/or children) who waited for access to an ambulatory or community outpatient service compared to the outcomes of those seen without delays. Three key concepts were included in the search strategy: “ambulatory care”, “delay” and “outcomes”.

Synonyms for ambulatory care (including broad terms such as “outpatients” and “community health” as well as particular services typically provided in these settings, such as “physiotherapy”) were searched and combined with the OR operator.

Due to the broad nature of the search terms required for the concepts of waiting and outcomes, a matrix of terms was used to search for papers that addressed outcomes of waiting (Table 1). The matrix combined terms such as ‘wait’, ‘prompt’, ‘delay’, and ‘timely’ with terms such as ‘outcome’, ‘impact’ and ‘consequence’, using proximity operators to find these terms where they occurred within the same phrase. For example, the search term “wait” and “impact” were combined with the relevant proximity operator in each database to find phrases such as “the impact of delay” or “does a long wait impact…”. Individual matrix searches were combined using the OR operator, and then with the ambulatory care search using the AND operator. See Appendix for search strategy.

**Table 1:** Terms used for search

P- People with a health condition, referred to an ambulatory and/or community service	I and O-Intervention and Outcome combined as per matrix
Ambulatory care or ambulatory services Outpatient clinics Community service* Community health Subacute or “sub acute” Allied health Multidisciplinary Developmental delay Physio* Occupational therap* Rehabilitation Physical therap* Speech pathology or speech language pathology Continen* Incontinen* Social work Chronic disease	Wait adj5 impact Wait adj5 consequence Wait adj5 effect Wait adj5 outcome Delay* adj5 impact Delay* adj5 consequence Delay* adj5 effect Delay* adj outcome Access adj5 impact Access adj5 consequence Access adj5 effect Access adj5 outcome “response time” adj5 impact “response time” adj5 consequence “response time” adj5 effect “response time” adj5 outcome “time to treatment” adj5 impact “time to treatment” adj5 consequence “time to treatment” adj5 effect “time to treatment” adj5 outcome Timely adj5 impact Timely adj5 consequence Timely adj5 effect Timely adj5 outcome
“P” terms combined with “or” in medline yielded 1,867,226	“I and O” terms combined with “or” yielded 7756 articles (medline)
“P” combined with “I and O” using “or” yielded 750 in medline.	
Same strategy applied through Embase, Psych Info and CINAHL yielded 3186	
With duplicates removed, final number of articles considered- 2327	

*signifies the search uses the word stem to find variations

Electronic data bases included in the search were Medline, Embase, Psych Info and CINAHL searched from the earliest available date until August 2017. To augment the search of electronic databases, reference lists of selected studies were scanned for relevant articles, and citation tracking of included papers completed in Google Scholar.

### Study selection

Articles were eligible for inclusion if they included patients accessing community outpatient services for a subacute or chronic condition, and provided data on comparative outcomes for patients who experienced delays in access to the service with patients who were seen more promptly. Only patient outcomes were considered, consistent with the definition of quality of care as “care that is clinically effective, care that is safe and care that provides a positive experience for patients” [21]. Therefore, patient outcomes included clinical outcomes, satisfaction, and health-related quality of life. Studies were excluded if they considered service factors only (for example attendance or dropout rates, resources provided or length of time in the service).

Only services that clearly provided community-based services for sub-acute or chronic conditions were included in this review. This was considered distinct from hospital outpatient departments or acute medical services. Therefore, studies were excluded if they described outpatient services for a specialised medical-only service (for example, surgical procedures, radiotherapy). Where a medical specialist was part of a multi-disciplinary team, such as a continence clinic or rehabilitation team including physician, the studies were included. The inclusion and exclusion criteria are described in Table 2.

**Table 2:** Inclusion and exclusion criteria

	Inclusion	Exclusion
Participants	• Clients/patients referred to and waiting for ambulatory or community health services.	• People waiting for: ○ Mental health/addiction services ○ Specialist medical services (eg. surgery, diagnostic services, radiotherapy etc). ○ Inpatient treatment ○ Case management services
Intervention or independent variable	• Waiting for an ambulatory/ community allied health/therapy service.	• Time spent in the waiting room/ED • Delay in seeking treatment • Factors associated with access to service not related to waiting (for example location /cost /knowledge /attitudes/insurance status /culture) • Studies with co-interventions. Eg Early intervention program involving high intensity treatment vs usual care.
Comparison	• People who waited vs people who didn’t wait for the same service • Comparison of outcomes cohorts of people who waited for different amounts of time for the same service	• Comparisons of different protocols to determine the optimum timing of a treatment • Studies without comparative data.
Outcomes	• Quality of care (safety, effectiveness, patient satisfaction) • Clinical outcomes • Satisfaction with care	• Service targets/outcomes/financial. • Patients/clients never seen/denied service through waiting • People who drop off the list having • waited/FTA • Drop out rate/non attendance/completion of program in terms of attendance • Satisfaction related to service processes
Publication type	• Journal articles • Qualitative and quantitative • Peer reviewed, with data • Case series • Published in English	• Conference papers/abstracts/thesis • Book chapters • Editorials/Opinion • Case study • Review

Titles and abstracts of identified studies were assessed independently by two reviewers against the selection criteria. Full text copies of articles were obtained for those that met selection criteria or where eligibility could not be established from abstract alone. Selection criteria were applied to full text articles independently by two researchers. Disagreements between researchers were discussed until consensus was reached. Interrater reliability was assessed using Kappa (κ) with 95% confidence levels with κ > 0.6 regarded as substantial agreement [22].

### Data extraction

Data extracted included: participant or patient group description; setting; study design; study quality; variable for comparison or correlation; reference waiting time (or magnitude of delay); and patient outcomes.

### Quality assessment

All study designs were accepted for review provided they met inclusion criteria. For this reason the McMaster Critical Review Forms [23, 24] as adapted by Imms [25] for qualitative and quantitative research were selected as an appropriate way to determine the methodological quality of each included study. These forms were used to assess quantitative studies on the basis of sample, measure and analysis and qualitative studies on the basis of credibility, transferability, dependability and confirmability. A star rating system required the reviewer to rate each criterion with one star for criteria not met; two stars for some evidence of criteria met; and three stars for evidence reported to meet criteria. Each study was therefore rated for quality out of a potential nine stars for quantitative and 12 stars for qualitative studies. Two reviewers independently scored the studies for quality. Scores were then compared and disagreements between researchers were discussed until agreement was reached and a final quality score allocated.

### Analysis

Study characteristics were explored for any associations or differences between treatment delays and patient outcomes. Clinically homogeneous data with a patient group, a common intervention/independent variable and outcome were synthesised descriptively. Where outcome measures were reported with sufficient detail, minimum clinically important differences (MCID) were calculated. Published MCID scores for improvement in metabolic equivalent [26], Patient Health Questionnaire 9 (PHQ9) [27], Roland Morris Disability Questionnaire [28] and Visual Analogue Scale [28] were utilised to determine clinical significance of results for these measures. For measures without published MCID, the MCID was estimated as half the control group standard deviation [29]. If the mean difference was greater than the MCID, the reported difference was considered to be clinically significant. Estimates of the mean and standard deviation were calculated for studies that reported median and ranges [30]. Where studies reported on more than two groups, the most extreme groups (i.e. the earliest and most delayed group) were compared for the purpose of assessing clinical significance. Studies that assessed differences between groups by responder analysis with variables dichotomised using a clinically meaningful threshold [31], were determined to have clinically significant results if participants who had a shorter wait time were significantly more likely to achieve this threshold.

The overall quality of the evidence and clinical impact of the results was determined utilising components of the National Health and Medical Research Council (NHMRC) body of evidence matrix [32]. The overall quality of the evidence was determined by assessing both the evidence base and consistency of findings. The evidence base and consistency of the evidence was assessed as high, moderate, low or very low. Where the evidence base consisted of randomised controlled trials with low risk of bias, the rating was higher (high, moderate). Where the evidence base consisted of cohort studies or studies with high risk of bias, the rating was lower (low, very low). Consistency was rated as high if all studies had consistent findings and very low if studies had inconsistent findings that could not be explained. No rating of consistency was calculated if only one study was included in the body of evidence. The lowest rating among studies included in the body of evidence, for either level of evidence or consistency, was used to assign the overall rating of the quality of the evidence. The clinical impact was determined by assessing the clinical significance of findings. Clinical impact was assessed as very large, substantial, moderate or slight dependent on the consistency of clinically significant results.

## Results

### Study selection

**Fig. 1:**
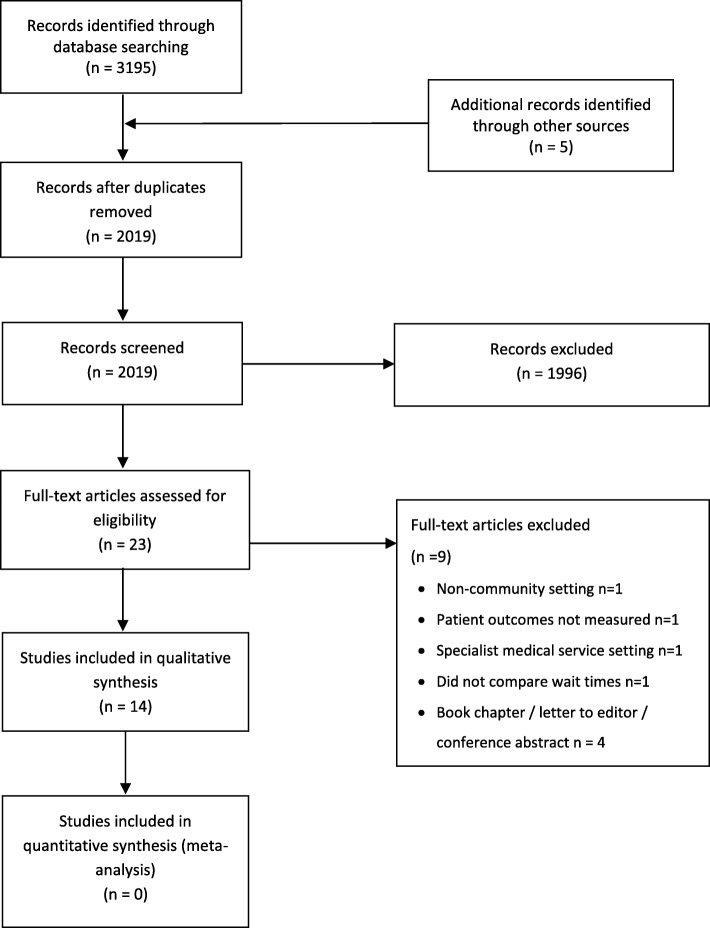
Article selection process

The data base search yielded 2014 articles with duplicates removed (Figure 1). Twenty-three articles were selected for full text review after screening of title and abstract. Of these, 10 were excluded due to: publication type (abstracts or book chapters, *n* = 4); [33–36]; comparison of different models or types of care rather than treatment delays (*n* = 3) [37–39] or not meeting inclusion criteria for service type (for example, medical only or inpatient care, n = 3) [40–42]. Reference and citation checks of the remaining 13 articles resulted in one additional article that met inclusion criteria [43], resulting in 14 included studies. The agreement between raters on study selection was substantial (κ = 0.64, 95% CI 0.35 to 0.93).

### Quality assessment

All but one of the 13 quantitative studies were rated as good quality [43–54], receiving at least 7 of 9 possible stars against the McMaster quality criteria, with the remaining study rated moderate in quality [55]. The only qualitative study included in this review was rated as good, receiving 10 of 12 possible stars [56]. The agreement between raters on the quality assessment was moderate (κ = 0.58, 95%CI 0.35 to 0.81).

### Study characteristics

#### Settings/population

The included studies involved a total of 69,606 adult participants. Sample sizes ranged between 22 [56] and 32,899 [46] and broadly described two major diagnostic groups: Musculoskeletal disorders including patients with mixed or non-specific musculoskeletal disorders [45, 49, 56], back pain [51–54] and hip fracture [55] (Table 3); and rehabilitation following a cardiac event [43, 44, 46–48, 50] (Table 4). Studies were completed in seven countries, with five from the United States of America (USA), two from Canada, two from Sweden, two from the United Kingdom (UK), and one each from Norway, Denmark and Australia.

**Table 3:** Study characteristics: Musculoskeletal

Author	Study quality	Setting	Participant/client group N	Study design	Waiting times compared	Outcome Measures	Key Findings
Amato et al. (1997) [45]	Sample^c^ Measure^c^ Analysis^a^	Orthopaedic rehab, USA	Musculo-skeletal disorders *N* = 24,196	Retrospective cohort study	Short wait: 0–7 days Long wait: ≥ 121 days	1. QOL 2. Patient Satisfaction	Authors conclude an association between shorter wait time and improved QOL but no difference in patient satisfaction.
Harding et al. (2013) [56]	Credibility^b^ Transferability^c^ Dependability^c^ Confirmability^b^	Outpatient musculo-skeletal rehab, Australia	Musculo-skeletal disorders *N* = 22	Qualitative component of a mixed methods study (semi-structured interviews)	Short wait: 10 days Long wait: 29 days	1. Patient experience	Patients who wait longer for first appointment report anxiety regarding physical deterioration.
Linton et al. (1993) [49]	Sample^c^ Measure^b^ Analysis^c^	Primary health care unit (GP with referral to Physical Therapist +/− case manager), Sweden	Musculo-skeletal pain *N* = 198	Controlled trial (non-randomised)	Short wait: 3 days Long wait: 9 days	1 Patient satisfaction 2. Pain 3. Workplace participation	Shorter wait time associated with improvement in workplace participation and patient satisfaction with short wait to first appointment. Wait time not associated with pain.
Nordemanet al (2006) [51]	Sample^c^ Measure^c^ Analysis^c^	Primary health care Sweden	Low back pain. *N* = 60	Randomised clinical trial	Short wait: within 2 days Long wait: 4 week delay	1. Pain 2. Function 3. Workplace participation	Wait time not associated with pain, function or workplace participation.
Pedersen et al. (2017) [55]	Sample^b^ Measure^b^ Analysis^b^	Municipal rehab, Denmark	Elderly patients post hip fracture. *N* = 116	Prospective cohort study	Median wait: 8 days Range: 0–64 days	1. Function	Wait time not associated with function.
Self et al. (2000) [52]	Sample^b^ Measure^c^ Analysis^b^	Ortho-paedic physical therapy, USA	Low back pain *N* = 161	Retrospective cohort study	Short wait: 0–7 days Long wait: 15–42 days	1. Function	Wait time not associated with function.
Wand, et al. (2004) [53]	Sample^c^ Measure^b^ Analysis^c^	Physio-therapy outpatient service, UK.	Acute low back pain. *N* = 102	Single blind randomised controlled trial	Short wait: 0–1 days Long wait: 42 days	1. Function 2. Pain 3. Anxiety symptoms 4. Depression symptoms 5. QOL	Shorter wait time associated with improved QOL and less anxiety and depressive symptoms. Wait time not associated with function or pain.
Zigenfus et al. (2000) [54]	Sample^c^ Measure^b^ Analysis^c^	Occupational health care/Physical therapy, USA	Workers with acute low back injuries. *N* = 3867	Retrospective cohort study	Short wait: 0–1 days Intermediate Wait: 2–7 days Long wait: 8–197 days	1. Workplace participation	Shorter wait time associated with improved workplace participation.

*QOL* Quality of life

^a^criteria not met

^b^criteria partially met

^c^criteria met in full

**Table 4:** Study characteristics: Cardiac rehabilitation

Author	Study quality	Setting	Participants/ client group. N	Study design	Waiting times compared	Outcome Measures	Key Findings
Aamot et al. (2010) [44]	Sample^b^ Measure^c^ Analysis^c^	Cardiac rehab program, Norway	Myocardial infarction *N* = 39	Randomised Control Trial: 2 groups	Short Wait: Immediate Long wait: 4 week delay	1. Exercise tolerance 2. QOL	Wait time not associated with exercise tolerance.
Fell et al. (2016) [46]	Sample^c^ Measure^c^ Analysis^c^	Outpatient cardiac rehab, UK	Acute coronary syndrome. *N* = 32,899	Retrospective cohort study	Short wait: 0–28 days Long wait: 29–365 days	1. Physical activity 2. QOL 3. Exercise tolerance	Shorter wait time associated with improvement in QOL and exercise tolerance. Wait time not associated with physical activity.
Johnson et al. (2014) [47]	Sample^c^ Measure^b^ Analysis^c^	Outpatient cardiac rehab, USA	Patients following interventions for cardiac disease. *N* = 1241	Retrospective cohort study	Short wait: 0–15 days Intermediate wait: 16–30 days Long wait: > 30 days	1. Exercise tolerance	Shorter wait time associated with improvement in exercise tolerance.
Kehler et al. (2017) [48]	Sample^b^ Measure^c^ Analysis^c^	Outpatient cardiac rehab, Canada	Cardiac events. *N* = 60	Prospective observational cohort study	Short wait: ≤ 60 days Long wait: > 60 days	1. Physical activity 2. Exercise tolerance 3. Depressive symptoms	Shorter wait time associated with improvement in exercise tolerance. Wait time not associated with physical activity or depressive symptoms.
Marzolini et al. (2015) [50]	Sample^c^ Measure^b^ Analysis^c^	Outpatient cardiac rehab, Canada	Post coronary bypass graft surgery. *N* = 6497	Retrospective cohort study	Short wait: ≤60 days Long wait: 241–365 days	1. Exercise tolerance	Shorter wait time associated with improvement in exercise tolerance.
Pack et al. (2013) [43]	Sample^b^ Measure^c^ Analysis^c^	Outpatient cardiac rehab, USA	Patients with non-surgical cardiac diagnosis. *N* = 148	Randomised controlled trial	Short wait: ≤10 days Long wait: 35 days	1. Exercise tolerance	Wait time not associated with exercise tolerance.

*QOL* Quality of life

^a^criteria not met

^b^criteria partially met

^c^criteria met in full

#### Study design

Studies included four randomised control trials [43, 44, 51, 53], one controlled trial without randomisation [49], six retrospective cohort studies [45–47, 50, 52, 54], two prospective cohort studies [48, 55], and one qualitative evaluation [56].

The studies either compared a group who waited with a group who received service with minimal delay [43, 44, 46, 48, 49, 51, 53] or compared multiple groups of people with different waiting times for treatment [45, 47, 50, 52, 54]. Three studies used regression analyses to investigate associations between waiting times and patient outcomes, either as the sole aim of the study [54] or in addition to between-group comparisons [45, 49].

#### Timing of intervention

The shortest difference between the comparison wait times was 1 to 2 weeks [49, 54, 55]. Four studies had a long difference in wait times between groups, ranging from 3 months to 12 months [45, 46, 50, 54] and five studies had a difference of approximately 1 to 2 months [43, 44, 51–53, 56]. Comparison in waiting time was not able to be determined in two studies [47, 48].

#### Outcomes

Twelve of the 14 studies included outcomes related to physiological well-being or functional performance. For those conducted with cardiac populations these included measures of exercise tolerance [43, 44, 47, 48, 50] and physical activity [46]. In musculoskeletal populations physical measures included function [51–53, 55] and pain [49, 51, 53]; and three of these studies also considered measures of workplace participation [49, 51, 54].

Three studies reported on anxiety or depressive symptoms [48, 53, 56]; three reported measures of quality of life [44, 45, 53] and three reported either patient perceptions of care [56] or measures of patient satisfaction [45, 49].

### The effect of waiting on patient outcomes

#### Musculoskeletal conditions

There was low to very low evidence suggesting that reduced wait times are associated with improvement in some outcomes for patients with musculoskeletal conditions (Table 5), but the clinical significance of these improvements was generally slight (Table 6).

**Table 5:** The impact of waiting for treatment for musculoskeletal conditions on health outcomes

Study	Outcome	MICD	Findings (Positive MD favours shorter wait)	Statistical Significance	Clinical Significance
Amato et al (1997) [45]	QOL (FOTO Outcomes Index)	Unable to estimate	Patients treated within 15 days had greater improvement in QOL	**?**	**?**
	Patient Satisfaction (FOTO Patient Satisfaction Index)	Unable to estimate	No association between patient satisfaction and waiting time	**N**	**N**
Linton et al (1993) [49]	Pain (Treatment outcome questionnaire)	Unable to estimate	No sig. difference between groups	**N**	**N**
	Workplace participation	≥ 1 day	History of MSP: No sig. differences	**N**	**N**
	Number of days off work each quarter		No History of MSP:		
			1^st^ Quarter: MD 11 (95%CI 0.01-22.0)	**Y**	**Y**
			2^nd^ Quarter: MD 7 (95%CI -2.7-16.7)	**N**	**N**
			3^rd^ Quarter: MD 11 (95%CI 2.8-19.2)	**Y**	**Y**
			4^th^ Quarter: MD 5 (95%CI -4.7-14.7)	**N**	**N**
	Development of chronic symptoms (proportion & RR)	Unable to estimate	History of MSP: No sig. differences	**N**	**N**
			No History of MSP: Short wait 2% vs. long wait 15%, RR 8.2 (95%CI 1.5-45.3)	**Y**	**?**
	Patient Satisfaction (Treatment satisfaction questionnaire)	Unable to estimate	Short wait group more satisfied with time to appointment (X^2^=15.8, *P*<0.01 with history of MSP, X^2^=9.4, *P*=0.02 with no history)	**Y**	**?**
			No sig. differences between groups in satisfaction with examination & treatment	**N**	**N**
Nordemann et al (2006) [51]	Pain				
	BRPP (change scores)	1.2 units	MD 0.10 (95%CI -1.0 to 1.2)	**N**	**N**
	ŐMPSQ (change scores)	11.7 units	MD 6.3 (95%CI -8.1 to 20.7)	**N**	**N**
	Function: RMQ (change scores)	3.5 units	MD -0.9 (95%CI -1.0 to 1.2)	**N**	**N**
	Workplace participation: ŐMPSQ (change scores)	1.1 units	MD -0.7 (95%CI -1.7 to 1.3)	**N**	**N**
Pedersen et al (2017) [55]	Function (SPPB)	Unable to estimate	*G* 0.10 (95%CI -0.1 to 0.2)	**N**	**N**
Self et al (2000) [52]	Function (TOAS)	Unable to estimate	No sig. differences between groups.	**N**	**N**
Wand et al (2004) [53]	Function (RMQ)	3.5 units	MD 1.8 (95%CI -0.4 to 4.0)	**N**	**N**
	Pain (VAS)	3.5 units	MD 0.9 (95%CI -0.04 to 1.8)	**N**	**N**
	Anxiety symptoms (STAIS)	2 units	MD 2.8 (95%CI 1.0 to 4.6)	**Y**	**Y**
	Depressive symptoms (MZDRS)	5.7 units	MD 8.4 (95%CI 3.9 to 12.9)	**Y**	**Y**
	QOL				
	EQ-5D Total Score	0.15 units	MD 0.10 (95%CI 0 to 0.2)	**N**	**N**
	SF-36 Physical Function	9.5 units	MD 3 (95%CI -4.8 to 10.8)	**N**	**N**
	SF-36 Role-Physical	21.5 units	MD 11 (95%CI -6.7 to 28.7)	**N**	**N**
	SF-36 Bodily Pain	11 units	MD 11 (95%CI 2.3 to 19.7)	**Y**	**Y**
	SF-36 General Health	9.5 units	MD 12 (95%CI 5.2 to 18.8)	**Y**	**Y**
	SF-36 Vitality	10.5 units	MD 22 (95%CI 13.7 to 30.3)	**Y**	**Y**
	SF-36 Social Functioning	12.5 units	MD 16 (95%CI 6.4 to 25.6)	**Y**	**Y**
	SF-36 Role-Emotional	21.5 units	MD 19 (95%CI 2.7 to 35.3)	**Y**	**N**
	SF-36 Mental Health	12 units	MD 22 (95%CI 13.5 to 30.5)	**Y**	**Y**
Zigenfus et al (2000) [54]	Workplace participation				
	Days away from work	≥ 1 day	Short wait vs. intermediate wait: MD 0.7 (95%CI 0.4 to 1.0)	**Y**	**N**
			Short wait vs. long wait MD 2.5 (95%CI 2.0 to 3.0)	**Y**	**Y**
	Days of restricted work duties	≥ 1 day	Short wait vs. intermediate wait MD 1.8 (95%CI 1.3 to 2.3)	**Y**	**Y**
			Short wait vs. long waitMD 5.3 (95%CI 4.4 to 6.2)	**Y**	**Y**

*MICD* Minimum Clinically Important Differences, *QOL* Quality of Life, *FOTO* Focus on Therapeutic Outcomes, *TOAS* Therapeutic Outcomes Assessment System, *BRPP* Borg Category Scale for Ratings of Perceived Pain, *ŐMPSQ* Őrebro Musculoskeletal pain Screening Questionnaire, *RMQ* Roland and Morris disability Questionnaire, *Sig* Significant, *SPPB* Short Performance Physical Battery, *VAS* Visual Analogue Scale, *STAIS* Spielberger State-trait Anxiety Inventory, *MZDRS* Modified Zung Self-Rated Depression Score, *EQ-5D* EuroQOL-5D, *SF-36* 36-item Short Form Survey, *MD* Mean Difference, *RR* Risk Ratio, *Y* Yes, *N* No; *?* Unable to determine

**Table 6:** Evidence synthesis for waiting for treatment for musculoskeletal conditions

Outcome	Number of trials	Number of participants	Overall effect of short wait	Level of evidence			Clinical impact
				Evidence base	Consistency	Overall	
Workplace participation	3 [49, 51, 54]	4125	Positive effect	Low	Low	Low	Moderate
Pain	3 [49, 51, 53]	360	No effect	Moderate	Excellent	Moderate	N/A
Function	4 [51–53, 55]	577	No effect	Moderate	Excellent	Moderate	N/A
QOL	2 [45, 53]	24,298	Positive effect	Moderate	Very Low	Very Low	Slight
Satisfaction	2 [45, 49]	24,394	Positive effect	Low	Very Low	Very Low	Slight
Depressive symptoms	1 [53]	102	Positive effect	Moderate	N/A	Moderate	Slight
Anxiety symptoms	1 [53]	102	Positive effect	Moderate	N/A	Moderate	Slight

*N/A* Not applicable, *QOL* quality of life

There was low-level evidence that short wait time may be associated with moderate improvement in workplace participation for patients with musculoskeletal conditions. This included reduced sickness absenteeism and days of restricted work duties [49, 51, 54]. Participants without a history of pain who had a shorter wait time took fewer days off work than those with a longer wait time [49]. There was moderate evidence that wait time was not associated with pain outcomes for patients with musculoskeletal conditions [49, 51, 53]. The three studies that measured pain in relation to waiting did not find statistically significant differences between groups in pain following completion of treatment. There were similar findings in relations to function [51–53, 55] and disability measures [51, 53], where there was also moderate evidence that these outcomes were not associated with wait time for this patient population. There was very low-level evidence that short wait time was associated with slight improvement in quality of life for patients with musculoskeletal conditions [45, 53]. Wand et al. [53] found a clinically significant difference in quality of life scores between patients randomly allocated to immediate and delayed treatment groups. Amato et al. [45] also observed improved quality of life for patients with shorter waiting periods in their retrospective cohort study, although the statistical significance of this finding was not reported.

There was very low-level evidence that short wait time was associated with slight improvement in patient satisfaction with wait time for patients with musculoskeletal conditions. There was no evidence that shorter wait time led to greater satisfaction with other aspects of care [45, 49].

There was very low-level evidence that short wait time was associated with slight improvement in depressive symptoms for patients with musculoskeletal conditions [53]. Harding et al’s qualitative evidence supports this finding; patients in this study who experienced delays in commencing rehabilitation reported negative psychological impacts, including feeling “demoralised” [56].

There was very low-level evidence that short wait time was associated with slight improvement in anxiety symptoms for patients with musculoskeletal conditions [53]. Patient anxiety about physical deterioration while waiting for therapy was reported in Harding’s qualitative study which supports this finding [56].

#### Cardiac conditions

Consistent with findings in musculoskeletal conditions, there was low to very low-level evidence indicating that reduced wait time for patients with cardiac conditions, referred to cardiac rehabilitation was associated with improvement in some patient outcomes (Table 7). The clinical significance of these improvements was generally slight (Table 8).

**Table 7:** The impact of waiting for cardiac rehabilitation on health outcomes

Study	Outcome	MICD	Findings (Positive MD favours shorter wait)	Statistical Significance	Clinical Significance
Aamot et al (2010) [44]	Exercise Tolerance (VO_2_ Peak)	3.1 ml/Kg per min	MD 0.1 (95%CI -5.2 to 5.4)	N	N
	QOL:				
	SF-36 General Health	4.4 units	MD -4 (-8.3 to 0.3)	N	N
	SF-36 Role Physical	12.5 units	MD -8.3 (95%CI -18.0 to 1.5)	N	N
	SF-36 Physical Functioning	8.1 units	MD 3.8 (95%CI -2.2 to 9.7)	N	N
Fell et al (2016) [46]	Physical Activity (Guideline adherence)	150 min/week	Long wait group: OR 0.9 (95%CI 0.7 to 1.0)	N	N
	Exercise Tolerance (Shuttle walk test)	≥70 m	Long wait group: OR 0.8 (95%CI 0.7 to 0.9)	Y	Y
	QOL (Dartmouth self-reported fitness)	1-3 (healthy status score)	Long wait group: OR 0.8 (95%CI 0.7 to 0.9)	Y	Y
Johnson et al (2014) [47]	Exercise Tolerance (MET change scores)	0.5 METs	Short wait vs. intermediate wait: MD 0.6 (95%CI 0.3 to 1.0)	Y	Y
			Short wait vs. long wait: MD 1.2 (95%CI 0.9 to 1.6)	Y	Y
Kehler et al (2017) [48]	Physical Activity (Guideline adherence)	150 mins/week	Short wait vs. long wait: 83% vs. 60%	N	N
	Exercise Tolerance (MET)	0.5 METs	MD 2 (95%CI 0.6 to 3.4)	Y	Y
	Depressive symptoms (PHQ-9)	5 units	MD 0.9 (95%CI -1.4 to 3.2)	N	N
Marzolini et al (2015) [50]	Exercise Tolerance (VO_2_ Peak)	8.5 ml/Kg per min	MD 14.5 (95%CI 10.0 to 18.1)	Y	Y
Pack et al (2013) [43]	Exercise Tolerance (MET)	0.5 METs	MD 0.1 (95%CI -0.3 to 0.5)	N	N

*MICD* Minimum Clinically Important Differences, *VO*_*2*_ *Peak* Peak Oxygen Consumption, *mL* milliliters, *Kg* kilograms, *min* minutes, *QOL* Quality of Life, *m* metres, *MET* Metabolic Equivalent, *PHQ-9* Patient Health Questionnaire – 9, *SF-36* 36-item Short Form Survey, *MD* Mean Difference, *OR* Odds Ratio, *Y* Yes, *N* No, *?* Unable to determine

**Table 8:** Evidence synthesis of impact of waiting for cardiac rehabilitation

Outcome	Number of trials	Number of participants	Overall effect of short wait	Level of evidence			Clinical impact
				Evidence base	Consistency	Overall	
Exercise tolerance	6 [43, 44, 46–48, 50]	40,884	Positive effect	Moderate	Low	Low	Moderate
Physical activity	2 [46, 48]	32,959	No effect	Moderate	High	Moderate	N/A
QOL	2 [44, 46]	32,938	Positive effect	Low	Very low	Very Low	Slight
Depressive symptoms	1 [48]	60	No effect	Low	N/A	Low	N/A

*N/A* not applicable, *QOL* quality of life

There was low-level evidence that short wait time may be associated with a moderate improvement in exercise tolerance in patients referred to cardiac rehabilitation [43, 44, 46–48, 50]. Three studies that considered this outcome were large retrospective cohort studies with a combined total of 40,637 participants, and all found clinically significant improvements in exercise tolerance for groups of patients who had shorter waiting time [46, 47, 50]. The findings were not replicated in the two randomised control trials that investigated exercise tolerance [42, 43].

There was moderate evidence that wait time was not associated with physical activity outcomes in patients referred to cardiac rehabilitation [46, 48]. Two studies found no difference between groups in self-reported achievement of the recommended guideline of 150 min/week of moderate to vigorous physical activity [46, 48].

There was very low-level evidence that short wait time may be associated with slight improvement in quality of life in patients referred to cardiac rehabilitation [44, 46]. Fell et al. [46] found a clinically significant difference between groups on the Dartmouth self-reported fitness measure.

There was low-level evidence that wait time was not associated with depressive symptoms in patients referred to cardiac rehabilitation [48].

## Discussion

There was low to very low-level evidence that a shorter wait time for community outpatient services may be associated with slight to moderate benefits for some patient outcomes. For patients referred to cardiac rehabilitation, there was low-level evidence to suggest that less wait time is associated with moderate improvement in exercise tolerance. For patients with musculoskeletal problems, there was low-level evidence suggesting that less wait time is associated with moderate improvement in workplace participation. Quality of life, patient satisfaction and psychological symptoms may also be positively influenced by short wait times, but further research is required to confirm this association. There is currently no evidence to suggest that the outcomes of pain, function or physical activity are better for patients who have more prompt access to care.

The benefits of reducing wait times may have been greater for services that had wait times of months or even a year compared to those that had wait times of days or weeks. For example, four of the six studies investigating the effect of delay in access to cardiac rehabilitation found a positive clinically important difference for those starting earlier [46–48, 50]. These four studies considered access delays ranging from “greater than 30 days” to up to 365 days. In contrast, the two studies that did not find a clinically important difference compared groups with wait times of less than 10 days with groups who waited 4 weeks; this could be considered a relatively small difference in wait times [43, 44]. Also clinical practice guidelines recommend that cardiac rehabilitation should start within 4 weeks from referral [57] so both groups in these studies [43, 44] adhered with best practice. It is possible that the benefits to patients of reduced wait times are greater where the baseline wait is very long or when clinical practice guidelines regarding timing of commencement of the service are not adhered to. Where the reduction in wait time is in months, rather than days and weeks, there may be greater benefit for patient outcomes [46, 50].

For patients awaiting cardiac rehabilitation, shorter wait times were associated with higher levels of exercise tolerance suggesting that prompt access to care may enhance the effectiveness of the intervention [58]. In this population, shorter wait times may influence patient outcomes secondary to the motivation for lifestyle change that many patients report immediately post cardiac event [59]. Specifically these patients may be more willing and motivated to make lifestyle changes and comply with exercise intervention when provided with the opportunity and support to do so during the early stages post cardiac event. The hypothesis that delays in access to care may miss an important window of opportunity for behaviour change is also supported by other studies that suggest that delays in access to cardiac rehabilitation are associated with a decrease in the rate of enrolments [60]. Positive behavioural changes are commonly demonstrated immediately after a traumatic event including a life threatening illness or injury [61]. It is speculated that this effect may translate to patients with other medical conditions such as people recently diagnosed with diabetes [62].

For patients with acute musculoskeletal pain, shorter wait times may be associated with reduced absence from work and a lower risk of developing chronic problems [49]. Musculoskeletal pain is associated with psychological impairment for the individual including depression, anxiety and sleep disorders [63]. Musculoskeletal pain is also a burden on the wider community in terms of productivity losses, and health, compensation or welfare costs [64, 65]. Improving patient participation at work can decrease the risk of ongoing disability [66] which along with receiving workers’ compensation are considered risk factors for development of chronic pain [67]. Therefore, reducing wait time for patients with acute musculoskeletal pain may be considered an important aspect of care if prompt treatment improves work participation, which may result in secondary benefits to the individual and society.

The impact of wait time for community outpatient services on patient outcomes such as quality of life and psychological symptoms was uncertain. Patients who experience long wait times for health care intervention such as elective surgery report psychological symptoms, including anxiety and depression [68] and it was anticipated that this review would find similar results. The evidence base was limited by the quality of the evidence and the relatively few studies that have measures these outcomes, suggesting that further studies are required to evaluate the effect of wait time on quality of life and psychological symptoms.

One interpretation of the results of this review of 69,606 patients in 14 studies is that waiting for community outpatient services only had a relatively small negative effect on some health outcomes. It is possible that the nature of the health conditions referred for community outpatient services means that waiting for these services does not affect disease progression. For example, many musculoskeletal conditions are characterised by resolution through natural history. Also, chronic conditions may not change over the course of a few months waiting. Perhaps the main benefit of reduced waiting for community outpatient services could be seen on health service factors, where the effect of waiting in one part of the system can cause bottlenecks in another, more critical, part of the system (for example emergency departments). Furthermore, managing wait lists can lead to inefficiencies within health services, as resources are redirected from frontline care into activities associated with organising the wait list such as triage, fielding phone calls and data management [11]. This negatively impacts on patients’ access to health services and ultimately reduces their overall quality of care [69].

Another potential benefit of reducing wait times that was not directly considered within this review was the experience of patients *during* the period-spent waiting. Although many patients may have a similar health outcome whether they receive a particular service after 1 month or 1 year, for some the 11 month delay may represent a period living in pain, with reduced participation in usual activities and resulting impacts on quality of life. Time off work, a factor found to be associated with access delays, was the only outcome considered in this review that reflected the experience *during* the waiting period, whereas other outcomes were measured at a time after treatment. A systematic review of the effect of waiting for treatment for chronic pain [70] further supports the hypothesis that long periods of time spent on wait lists prior to treatment has negative impacts; they concluded from 18 controlled trials that investigated the experience of patients on wait lists for chronic pain treatment that waits of 6 months or more for treatment for chronic pain were associated with deterioration in health-related quality of life, psychological well-being and depression. It is also of note that, consistent with this review, associations between wait time and outcomes *following* treatment for this population were limited and inconclusive [70]. While what happens to patients both *during* and *after* waiting is important it could be argued that the emphasis of this review on *after* reflects the effect of waiting on the endpoint and final outcome of patient care.

This review was registered prospectively and PRISMA guidelines were followed. The broad nature of the topic presented a search challenge. Terms such as “waiting” and “outcomes” could not easily be searched without producing an excessive and unmanageable yield of articles. This was addressed by designing a “matrix strategy” to capture the concept of “impact of waiting”. This provided a feasible method for searching an otherwise challenging question, but it is possible that some papers may have been missed. However, given that only one additional article was identified through checking of citations and abstracts it appears that the strategy captured relevant literature. We acknowledge that some large bodies of literature that may contribute important insights into this topic were excluded from this review, particularly in the areas of mental health services and specialist medical services, including surgery. It is possible that a long wait time may negatively affect dropout rate, adherence to treatment and non-attendance, which could impact on patient outcomes. These service-related outcomes were excluded as they are not directly related to patient outcomes. It is also noted that the search yielded studies from two diagnostic areas and it is thought that there would be impacts of waiting for other community outpatient services such as paediatric developmental therapy, continence services and many others. All studies were conducted in high income countries and therefore generalisability to lower-middle income countries may be limited. It is also noted that studies did not investigate the influence of seasonal factors (e.g. month of referral/first scheduled appointment) on waiting time, which may have influenced the results of this review. To improve the generalisability of these results, further research is suggested in populations with a variety of health conditions; in countries that are low-middle income; in medical-only clinics and qualitative research on the experience of patients while they wait. A systematic review on the impact of waiting on dropout rates, compliance and attendance at outpatient clinics is also suggested.

## Conclusion

This review found low to moderate levels of evidence, to suggest an association between early access to community outpatient services and improvement of some patient outcomes for those with cardiac conditions and musculoskeletal pain. Specifically, shorter wait times for cardiac rehabilitation may improve patient exercise capacity. Shorter wait times for musculoskeletal pain services may improve work participation. The effects of a short wait time for other patient conditions and patient outcomes, including quality of life, psychological symptoms and patient experience, are inconclusive.

## Abbreviations

CI: Confidence Interval
k: Kappa
MCID: Minimum clinically important differences
NHMRC: National Health and Medical Research Council
PHQ: Patient health questionnaire
PRISMA: Preferred reporting items for systematic reviews and meta-analyses
UK: United Kingdom
USA: United States of America
